# Disposal Practices of Unused and Expired Medicines Among the General Public and Pharmacies: A Mixed-Method Study in the Odisha State of Eastern India

**DOI:** 10.7759/cureus.52359

**Published:** 2024-01-16

**Authors:** S Suneeti Kanyari, Tapas Ranjan Senapati, Ansuman Kar

**Affiliations:** 1 Community Medicine, Kalinga Institute of Medical Sciences, Bhubaneswar, IND

**Keywords:** mixed-method study, odisha, medication disposal, unused medicines, expired medicines

## Abstract

Introduction

Unused and expired medicines are potentially toxic substances that should be managed effectively to avoid possible environmental hazards. The absence of well-defined protocols in India regarding the proper disposal of unused, unwanted, and expired medications raises concerns about the potential for accidental poisoning and environmental threats. Consequently, this research was conducted to evaluate the disposal practices of medications within both urban slum households and pharmacies.

Methods

This questionnaire-based, mixed-method study was conducted among 385 general public residing in urban slums and 10 registered pharmacies in Bhubaneswar city, India, from September 2020 to August 2021. A semi-structured questionnaire was framed to collect data from the households and the pharmacies; key informant interview was carried out among expert members regarding the disposal of expired medicines.

Results

Results found that 82.1% (316/385) of the study population threw unused drugs in dustbins, whereas only 2.6% (10/385) returned the medicines to the pharmacy. The most common reason for leftover medicine was the stoppage of drugs on symptomatic relief (53.2%, 205/385), followed by changes in prescription by doctors (18%, 69/385). Out of 10 pharmacies, seven pharmacies disposed of expired drugs in general dustbins.

Conclusion

Our findings indicate that a majority of the participants tend to dispose of medicines in an unsafe manner. Conversely, the responsible disposal of medications is significantly influenced by patient education. Hence, healthcare professionals are better positioned to impart knowledge to the public, fostering awareness about the proper storage and safe disposal of unused, unwanted, and expired medicines.

## Introduction

Pharmaceutical products have been used in increasing quantities globally [1]. Data from the World Health Organization (WHO) reveal that more than half of all medications are prescribed and sold inappropriately, resulting in the unnecessary accumulation of medicines and posing a significant environmental threat [2]. Factors such as poor patient adherence to medication, excessive prescribing by physicians, early resolution of medical conditions, or alterations in therapy regimens contribute to the wastage of medications [3,4]. The presence of unused and expired medications in homes and communities poses risks to humans, the environment, and other ecological species [5,6]. Two illustrative examples highlight the consequences of this issue: a) the exposure of the non-steroidal anti-inflammatory drug (NSAID) diclofenac, leading to renal failure and the near extinction of vultures in South East Asia [7]; and b) the exposure of the oral contraceptive pill ingredient ethinylestradiol (EE2), resulting in impaired sexual development and feminization of fish in European waters [8].

India holds the fifth position in terms of volume production in the global pharmaceutical markets [2]. The pharmaceutical business in India encompasses the manufacturing of drugs, finished dosage production, excipients synthesis, and the analysis of impurities and raw material evaluation [3,4]. Research indicates that a considerable portion of these pharmaceutical products remains unused or expired [5-7]. According to the WHO, 50% of medicines are prescribed, dispensed, or sold inappropriately, and half of all patients do not take them as directed [8]. Consequently, when these medications are not consumed correctly, they end up being left unused and wasted.

The accumulation of leftover, unused, or expired medications in households, along with their improper disposal, poses a significant threat to environmental contamination. This accumulation can result from various factors, such as changes in prescribed drugs, adjustments in drug dosage, medication expiration, or recovery from illness [9-12]. Another common practice among the general public is storing medicines at home for potential future use [13,14].

The expiration of drugs can result in a loss of efficacy, potency, and safety, leading to the formation of products that pose health risks. The ultimate outcomes of expired drugs may include antibiotic resistance, therapeutic failure, and potential carcinogenic effects. Additionally, expired drugs can contribute to accidental poisoning, drug abuse, the inefficient use of healthcare resources, and missed opportunities for medical treatment. The storage of unused or expired drugs may also alter their physico-chemical properties, promote moisture absorption, and consequently induce microbial contamination. It is crucial to enforce stringent compliance with disposal practices for unused or expired drugs [9,10].

Effective disposal practices for unused and expired drugs at the household level involve carefully containing the medications with other inert substances before delivering them to a pharmaceutical center for destruction [11,12]. While incineration is considered the optimal disposal method for medications, it is not practical at the household level [6,13]. Notably, existing international guidelines, such as those from the WHO, are primarily designed for national authorities such as the Ministries of Health, Ministries of Environment, and Drug authorities, overlooking the household level [14,15]. The available international guidelines on proper healthcare waste disposal methods are either inadequately adapted for public use or are largely unknown to the general population [14]. There is a pressing need to educate households about appropriate storage and disposal practices for unused and expired medications [13]. However, the implementation of proper disposal practices encounters numerous challenges, including the absence of standardized drug disposal protocols, specifically addressing household medications, some pharmacies refusing to accept unused and expired medications or discouraging such practices, and insufficient reinforcement of international guidelines [13,16,17].

There is a dearth of information regarding the disposal practices of unused and expired medicines at the household level in the study area. Therefore, it is crucial to examine the disposal practices of medications in both households and pharmacies, considering the significant impact it holds. The findings from such an assessment are essential for informing policy decisions and guiding the implementation of appropriate measures.

## Materials and methods

Study design and setting: This questionnaire-based, mixed-method study was conducted among the general public residing in the field practice area of Urban Health and Training Centre (UHTC), Kalinga Institute of Medical Sciences (KIMS), Bhubaneswar, India, from September 2020 to August 2021. Ten registered pharmacies of the city located in and around the study area were also included in this study.

Study population: This study encompassed individuals of all genders aged 18 years and above, residing in the above-mentioned study area, with no specified upper age limit for inclusion in the study.

Inclusion criteria: The inclusion criteria for the study comprise individuals with exposure to medicines during the previous three months, who are willing to share information, with a minimum age requirement of 18 years. Additionally, registered pharmacies in Bhubaneswar city are considered eligible participants.

Exclusion criteria: Exclusion criteria included persons not willing to share the information and not willing to participate in the study.

Sample size: Assuming p=50% (50% of the general public lack proper knowledge regarding safe disposal of unused medicines) as previously no similar study had been conducted in this area. With absolute precision d=5% with a 95% confidence interval, we calculated the sample size to be 385 from the general public.

For the qualitative part, 10 registered pharmacies of Bhubaneswar city located in and around the study area were chosen.

Study tool: The questionnaire comprised two parts. The initial part focused on gathering personal details of the study participants, such as gender, age, marital status, education, occupation, and number of family members. The second part delved into the practices and perceptions of the respondents regarding the disposal of unused medication. Questions were framed in English and translated into the vernacular language Odia in simple terms appropriate for the level of education of the consumers. The questionnaire was validated, and a pilot testing involving 30 respondents was conducted to further refine the questionnaire.

Methodology: The study area consisted of different slums arranged in parallel lanes. The households in each lane of the study area were visited consecutively, and any one member of the household, above 18 years of age, present during the time of visit, was considered a study participant. If any household was locked on the day of the visit or no adults were present at the time of the visit, we skipped that house and moved to the next household. The purpose and importance of the study were explained to the individual before filling out the questionnaire. After obtaining written informed consent, the structured questionnaires were distributed to them, and enough time was provided to fill out the questionnaire. For illiterate study participants, the interviewer read out the questionnaire and options before them and recorded their responses. For the registered pharmacies, a key informant interview was carried out among expert members regarding the disposal of expired medicines.

Statistical analysis: Data were entered and analyzed using Statistical Product and Service Solutions (SPSS, version 21) (IBM SPSS Statistics for Windows, Armonk, NY). Descriptive statistics was used to demonstrate the different variables. The quantitative data were computed as percentages and presented in tables. The qualitative data obtained from key informant interviews were transcribed verbatim, translated where necessary and notes were made.

Ethical consideration: The study has been approved by the Institutional Ethics Committee, Kalinga Institute of Medical Sciences, bearing number KIIT/KIMS/IEC/394/2020.

## Results

Table 1 depicts the sociodemographic characteristics of the study participants, of this 57% (219/385) were males and 43% (166/385) were females, mainly from the age group of 18-40 years. Meanwhile, 83% (320/385) of the study participants were married, and 47% (181/385) had maximum education up to primary school. Additionally, 89% (343/385) were employed. The detailed stratification of the kind of occupation was beyond the scope of the study.

**Table 1:** Sociodemographic characteristics of the study population (N=385)

Demographic parameters		Number (n)	Percentage (%)
Age	18-40 yrs	223	58
	41-60 yrs	112	29
	≥ 61 yrs	50	13
Gender	Male	219	57
	Female	166	43
Marital status	Married	320	83
	Unmarried	65	17
Education	Illiterate	50	13
	Upto primary school	181	47
	Upto secondary school	119	31
	Intermediate or higher	35	9
Occupation	Unemployed	42	11
	Employed	343	89
Total no. of family members	≤5	293	76
	6-10	92	24

Table 2 depicts the common types of medicines that are unused or expired in the study population. Analgesics (68.8%, 265/385) were the most common class of unused or leftover medicine, followed by antacids (67%, 258/385), antibiotics (58.2%, 224/385), vitamins (56.1%), and cough and cold medicines (33.8%, 130/385). Fifty-five (14.2%) participants had other classes of leftover medicines such as antihypertensive drugs, antidiabetic drugs, and antifungal drugs.

**Table 2:** Common classes of leftover medicines in the households of the study population

Class of drugs	Number (n)	Percentage (%)
Analgesics	265	68.8
Antibiotics	224	58.2
Topical antibiotics	39	10.1
Antidiarrhoeals	103	26.8
Antacids	258	67
Vitamins	216	56.1
Cold & cough medicines	130	33.8
Others	55	14.2

In Table 3, the reasons for storage/leftover of unused drugs at home were assessed. In this study, the most common reason for leftover medicine was the stoppage of drugs on symptomatic relief (53.2%, 205/385), followed by changes in prescription by doctors (18%, 69/385) and non-compliance to therapy (14.5%, 56/385).

**Table 3:** Reasons for leftover medicines at home

Reasons	Number (n)	Percentage (%)
Changes in prescription by doctors	69	18.0
Prescribing more no. of drugs than required	21	5.5
Stoppage of drugs on symptomatic relief	205	53.2
Non-compliance to therapy	56	14.5
Purchase more drugs expecting future need	34	8.8

Table 4 depicts the disposal practices of unused medicines, followed by the study population where 82.1% (316/385) interviewees threw the unused drugs in the dustbin, 6.2% (24/385) flushed in the toilet, 4.2% (16/385) buried in the land, and only 2.6% (10/385) returned the medicines to the pharmacy.

**Table 4:** Practice of disposal of leftover drugs

Method of disposal	Number (n)	Percentage (%)
Return to pharmacy	10	2.6
Land burial	16	4.2
Flush in toilet	24	6.2
Burn	7	1.8
In dustbin	316	82.1
In rivers or water bodies	8	2.1
Donation to poor	4	1.0

Table 5 describes the outcomes of the key informant interview among pharmacies. Seven out of 10 pharmacies dispose of the expired drugs via general dustbins. Eight pharmacies felt that the distributor or the company should take back the expired drugs. Additionally, 50% (5/10) of the pharmacies ensured proper storage and segregation of expired drugs, and 60% (6/10) felt that there was a need for the introduction of pharmaceutical waste management in the curriculum of the students.

**Table 5:** Key informant interview outcomes of the pharmacies (N=10)

Question Category	Responses	Description
What are the various ways expired drugs are disposed of in Bhubaneswar city?	Through: General Dustbins (7), Burnt (2), Returned to the Company (1)	Many dispose of via general dustbins - Some dispose of by burning the drugs - Few others return it to the distributor.
Should drug distributors or wholesalers collect back their expired drugs?	Yes (8), No (0), Can’t Say (2)	The majority of pharmacies felt that the distributor or the company should take the expired drugs back.
Do pharmacies ensure proper storage and segregation of expired drugs?	Yes (5), Sometimes (3), No (2)	Fifty percent of pharmacies said yes; however, another 50% either practiced segregation sometimes or never had any type of segregation.
Is there a need for a drug disposal policy in India?	Yes (7), No (2), Can’t Say (1)	The majority felt there was a need for a drug disposal policy, while others either had no knowledge or felt no need for any change.
What are the common challenges for the proper disposal of expired drugs?	-	Top on the list: Poor knowledge about drug disposal, lack of proper law enforcement and public advisory, lack of facilities for proper disposal, Others on the list, lack of training of health professionals about disposal.
What are your recommendations?	-	Top on the list: Public awareness and relay of drug disposal, penalty to distributors for non-compliance, training and education of pharmacies.
Will you approve lectures on pharmaceutical waste management classes in schools of pharmacy?	Yes (6), No (2), Can’t Say,(2)	The majority felt there was a need for the introduction of pharmaceutical waste management in the curriculum of the students.

## Discussion

This study was carried out among 385 individuals, including 57% males and 43% females. A study conducted in Kabul with a sample of 301 had 73.4% males and 26.6% female respondents. More than half of the interviewees were university graduates [18]. Another study in Saudi Arabia, Wajid et al. [19], had 40.7% of married respondents, and 74.7% of interviewees were university graduates. In India, a study was conducted among patients visiting OPDs in Western India [1]; 77.5% of the respondents were male, the mean age was 32±6.2 years, and the consumers were mainly from middle to lower socio-economic strata [1].

This study found that analgesics (68.8%) were the most common class of leftover drugs in households. In a study conducted in Kabul, the prevalence of unused medicines was 95.3%, of which 46.5% of antibiotics and 20.3% of NSAIDS were mostly stored at home and then disposed of [18]. A Saudi study revealed the prevalence of unused drugs is 89.3%, of which 80.7% of leftovers are NSAIDs and 48.7% are antibiotics [19]. In India, a study conducted in Ahmedabad showed that 30.5% of analgesics, 17.5% of vitamins, and 11% of oral and topical antimicrobials stored at homes were unused and later disposed of [1]. The reason for the storage of drugs was attributed to stoppage of symptomatic relief (29%), frequent change in prescription (20%), prescription of more drugs than required (10%), and some other reasons.

In Kabul, a study found that 52.2% kept unused medicines at home until expiry, followed by 21.3% returning to a medical store and 14.3% throwing them in dustbins. However, 77.7% of expired medicines were thrown in dustbins, followed by 12% flushed into the toilet [18]. Similarly, in Saudi, 48.1% threw away unused drugs in the garbage, 13.7% gave them to friends or relatives, and 5.4% flushed the medicines into the toilet [19]. In Western India, a study found that 30.5% of consumers threw unused medicines in dustbins, 12% flushed them in the toilet, and 8% returned them to the pharmacy [1]. Additionally, 60.8% of respondents in Kabul and 85.5% of respondents in Saudi felt the need for the government to create awareness programs for safe disposal of expired/unused medicines.

Overall, a majority, exceeding 90%, of participants in the study area indicated that they had not been informed about the correct and safe methods of disposing of medications. Consequently, it is imperative for healthcare providers to implement educational initiatives addressing the proper and safe disposal of medications, ensuring that patients receive this crucial information during their visits to healthcare facilities. Notably, healthcare providers play a significant role in disseminating accurate information regarding the safe disposal of medications.

The current priority lies in prioritizing the take-back initiative program as the first step, followed by raising public awareness about the repercussions of existing disposal methods on both personal health and the environment. This approach has the potential to yield positive outcomes in mitigating the prevailing disposal practices.

A limitation of this study is that the study area was limited to specific locations within the city of Bhubaneswar. Another limitation may be recall bias as we included study participants with exposure to medicines in the last three months. A more extensive survey that includes remote areas could uncover additional valuable information.

## Conclusions

Among our study population, 97.4% disposed of unused drugs improperly, whereas only 2.6% returned the expired drugs to the pharmacy. Improper disposal of unused and expired medicines is becoming a global problem, especially in developing countries such as India. It is much more because of poor regulation of the health sector, and a significant part of the population is less aware of the drug disposal policies and hazards associated with their improper implementation. Addressing the issue of unused and expired medicines in India requires a multifaceted approach involving individuals, healthcare providers, pharmaceutical companies, and government bodies to ensure efficient resource utilization, reduce waste, and safeguard public health and the environment.
